# The interplay between seasonality and density: consequences for female breeding decisions in a small cyclic herbivore

**DOI:** 10.1186/1472-6785-14-17

**Published:** 2014-05-28

**Authors:** Adrien Pinot, Bertrand Gauffre, Vincent Bretagnolle

**Affiliations:** 1 Centre d’Etudes Biologiques de Chizé (CEBC-CNRS), Beauvoir sur Niort 79360, France; 2 Clermont Université, VetAgro Sup, BP 10448, Clermont-Ferrand F-63000, France; 3 INRA, USC1339 (CEBC-CNRS), Beauvoir sur Niort F-79360, France

**Keywords:** Seasonality, Population cycles, Common vole, Density-dependence, Fecundity

## Abstract

**Background:**

Cyclic rodent population dynamics are subjected to both intrinsic regulatory processes such as density-dependence and extrinsic environmental forcing. Among extrinsic factors, seasonal environmental variation is understood to facilitate cycles. In rodents, these processes have been studied mostly independently and their relative importance for population dynamics is poorly known.

**Results:**

We performed a detailed analysis of common vole (*Microtus arvalis*) reproduction in a cyclic population using a spatially extensive data set over 17 years in central-western France. Environmental seasonality was the main source of explained variation in common vole reproduction. Additionally, inter-annual variation in the environment explained a smaller part of the variance in reproduction in spring and summer than in winter, whereas the effect of density was only found in autumn and winter. In particular, we detected a strong impact of plant productivity on fecundity during the breeding season, with low vegetation productivity being able to bring vole reproduction nearly to a halt. In contrast, vole reproduction during autumn and winter was mainly shaped by intrinsic factors, with only the longer and heavier females being able to reproduce. The effect of population density on reproduction was negative, mediated by direct negative effects on the proportion of breeders in autumn and winter during outbreak years and by a delayed negative effect on litter size the following year.

**Conclusions:**

During the main breeding season, variability of female vole reproduction is predominantly shaped by food resources, suggesting that only highly productive environment may induce vole outbreaks. During fall and winter, variability of female vole reproduction is mainly controlled by intrinsic factors, with high population density suppressing reproduction. This suggests, in this cyclic population, that negative direct density dependence on reproduction could explain winter declines after outbreaks.

## Background

Demographic rates such as immigration, emigration, birth and death depend on density-independent (i.e. environmental factors) as well as density-dependent processes, which ultimately cause changes in population size [1]. At temperate and arctic latitudes in particular, seasonal fluctuations in climate and day length constrain the primary production of ecosystems. For animals, this seasonality of environment constrains reproduction both in the timing [2] and duration of the breeding season [3]. Indeed, variation in day length has been found to have a major impact on several life history traits related to breeding (reviewed in [2]). Population dynamics of herbivores is also severely impacted by food availability [4-6]. This is particularly true for rodents, which are small species with high fecundity, and are therefore highly sensitive to changes in food availability, notably through the regulation of reproduction. Food quality and quantity have been shown to accelerate rodent sexual maturation [7,8], to increase the length of the breeding season [9,10], the fraction of the population breeding [8-10] and litter size [11,12]. Additionally, population density may act negatively on reproduction when population size approaches or exceeds the carrying capacity (e.g., [13,14]), through an increase in competition (interference or resource depletion [15]).

The role of density dependence is particularly important in cyclic populations [16-18] and reproduction is known to vary with the phase of the cycle (*e.g.*[19-21]). Density dependent reproduction rates are actually suspected to play a leading role in the crash phase, as observed in some cyclic systems [21,22] and as predicted mathematically [23]. Indeed, Smith et al. [23] and Ergon et al. [21] suggested that a delayed density-dependent effect in the timing of the onset of breeding was sufficient to generate population cycles in the field vole *Microtus agrestis* in the UK. Conversely, density-independent factors have been less studied in cyclic systems, despite the fact that most cyclic rodent populations are found in highly seasonal environments [24]. Even fewer studies have investigated simultaneously the density-dependent (intrinsic) and density independent (extrinsic) influences on cyclic dynamics (but see [25,26]), although such joint analyses are crucial to understand variation in complex population dynamics, since failure to account for one type of effects may bias estimates of the other.

Several studies have separately investigated the intrinsic and extrinsic population processes in populations of the common vole (*Microtus arvalis*), but their combined effect on the population dynamics are still poorly understood. This species is well known for its episodic plagues [27,28], and exhibits cyclic dynamics in many parts of Europe [28-31]. Locally, densities of up to 2000 individuals per hectare have been described [32,33] at which negative density dependence is expected to occur. Furthermore, other intrinsic factors, such as kinship [34], weight [35] or age [36,37] have been demonstrated to impact reproduction. The common vole is also a seasonal breeder [38,39] with a higher percentage of breeders and larger litter size in spring and summer than in winter [35,40-43]. Several environmental parameters are known to affect breeding, such as photoperiod [44-46], temperature [47,48] and food quality [34,38,40].

**Figure 1:**
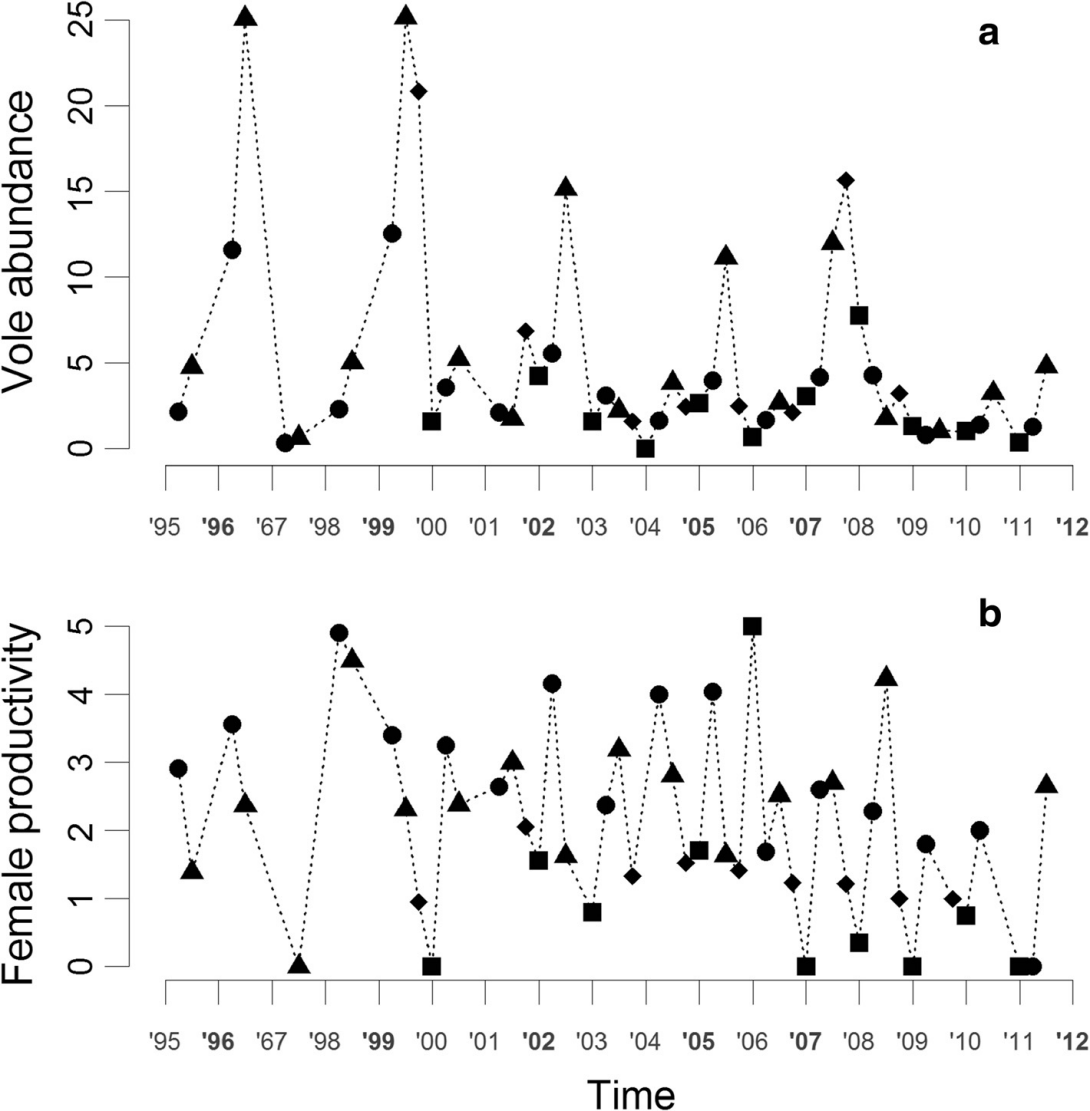
**Vole abundance and reproductive effort time series.** Times series of common vole abundance **(a)**, units; number of vole catch per 100 traps) and common vole female per capita productivity **(b)**, units; number of embryos per females) calculated at the study area level for each season. Seasons are defined as spring (circles; 16th February-15th May), summer (triangles; 16th May-15th August), autumn (diamonds; 16th August-15th November) and winter (squares; 16th November-15th February).

In a previous study of common voles in central-western France, Inchausti et al. [26] showed that the life history traits related to reproduction were not regulated by direct density dependent processes. Only a slight negative delayed density effect was observed on litter size and the proportion of breeders, particularly in the spring following a year of peak densities. However, this study focused on a restricted part of the year (April to June) and took into account only intrinsic factors in the analysis. Using the same data set (now extended to 17 years), we performed a more detailed analysis taking into account both the seasonal environment (i.e. intra-annual variation in day length, temperature and an index of plant productivity) and its interaction with density. We expected a strong seasonal pattern of reproduction due to the predictable seasonality of extrinsic variables but, in addition, we predicted that inter-annual variability in the environment would impact the reproduction rate. We take advantage of the considerable variation in density along the vole cycle (Figure 1a) to analyse the extent to which the timing of the onset and end of the breeding season result from an interaction between seasonality and density (see [17,23] for studies conducted on the close relative *M. agrestis*). Focusing on common vole female productivity, our aims were i) to assess accurately the role of environmental variability on reproduction, by decomposing seasonality into its predictable component (mean multi-annual pattern) and the inter-annual deviation from the mean (i.e., season quality); ii) to analyse the contributions of extrinsic and intrinsic factors in determining reproductive output, and iii) to empirically explore the existence of a delayed density-dependent on reproduction. Our analysis is based on a time series of 17 years (Figure 1b).

## Methods

### Study area

The study was conducted at the LTER “Zone Atelier Plaine & Val de Sèvre” (see [43,49]) located in western France (46°2’N, 0°4’W) and covering 450 km^2^ of intensive agricultural landscape. Land use is dominated by winter cereals (42.03%), sunflower and maize (22.22%), rapeseed (8.88%), grassland (8.36%) and alfalfa (4.74%; all data from 2011).

### Vole sampling

We used the time series data already published in Inchausti et al. [26] and Lambin et al. [31], extended with data obtained since then, but with a slightly different capture design (see below). Until 2010, the trap lines were set active and checked after 24 hours, and consisted of a 100-m transect with 51 unbaited single capture live traps spaced every 2 m, following a standardized protocol developed by Spitz [50] that eventually became the standardised protocol all over France. This protocol was in full compliance with the French law until 2012, in which the common vole was considered as a pest that should be destroyed by any means [28,51-53] because of outbreaks that caused crop damage [27,51-53] with important financial cost [28]. In 2012, following the directive 2010/63/UE (22/9/2010) issued by European parliament, a “*Comité d’éthique en matière d’expérimentation animale*” (CEMEA) has been launched locally, and will be in charge of evaluating trapping procedures. Since 2010 however we modified our long term trapping protocol in order to maximize survival and welfare of individuals and comply with future animal welfare policies to be introduced in France in 2013. The spatial sampling design and traps were identical (in order to keep the time series unaffected) but we added a second chamber to the trap (a plastic box) in which wood shavings and food (wheat and carrot) were provided, as in [54]. Manipulation time of live animals has always been kept to a minimum, and all live animals have been released at the location of trapping.

The sampling of common vole populations consisted of two-weeks long trapping sessions carried out at least twice a year from April 1995 to April 2011. A total of 4573 trapping lines were settled during this period, with about half the sampling effort concentrated in two sessions: April and June (respectively 29.1% and 24.1%). Sampling was restricted to April and June until 1999, after which additional trapping sessions were performed, less regularly (i.e., the same months were not sampled every years; see Tables 1 and 2 for trapping effort and sample sizes for each month and year). Despite an important sampling effort, we acknowledge that the heterogeneity of data across years and months weakens to some extent our analyses. Nine sectors of similar size were defined for logistical convenience. In each sector and trapping session, 10 field trapping lines were selected semi-randomly from a map in a regular design along a transect crossing the sector, sampling all the major crop types (but excluding unsuitable bare ground and over-sampling alfalfa and grasslands relative to their availability in the landscape for their role as potential vole refuge or reservoir year-round). Trapping lines locations changed between sessions.

**Table 1:** Sampling effort: Number of trapping line per year and months

	**1995**	**1996**	**1997**	**1998**	**1999**	**2000**	**2001**	**2002**	**2003**	**2004**	**2005**	**2006**	**2007**	**2008**	**2009**	**2010**	**2011**	**Sum**	**%**
**jan**									20				20	10				50	1,09
**fev**						20		20	20	20	20	30		50	39	40	34	293	6,41
**mar**						20	20	20	20		30	50	20	40	37	26		283	6,19
**avr**	132	96	96	80	80	100	40	120	70	100	89	80	80	40	90	10	27	1330	29,08
**may**	12						50	1	30	20		50	30	50			30	273	5,97
**jun**	51	88	96	80	40	100	90	60	40	80	100	60	90	68			58	1101	24,08
**jul**	93	8			40		20	60	40		20	70	40	20	88	4	29	532	11,63
**aug**					20					10	20	10	40	80				180	3,94
**sep**					20		20				20	10						70	1,53
**oct**					21				20	20	20	20	40					141	3,08
**nov**							20			20	20	20		40	40			160	3,50
**dec**										20		20	40	40	40			160	3,50
**Sum**	288	192	192	160	221	240	260	281	260	290	339	420	400	438	334	80	178	4573	100
**%**	6,30	4,20	4,20	3,50	4,83	5,25	5,69	6,14	5,69	6,34	7,41	9,18	8,75	9,58	7,30	1,75	3,89	100	

**Table 2:** Sampling effort: Number of individual on which analyses are based per year and months

	**1995**	**1996**	**1997**	**1998**	**1999**	**2000**	**2001**	**2002**	**2003**	**2004**	**2005**	**2006**	**2007**	**2008**	**2009**	**2010**	**2011**	**Sum**	**%**
**jan**									5									5	0,31
**fev**						2		9	7					32	1	3	1	55	3,38
**mar**						2	3	8	2		1	3		13	8	2		42	2,58
**avr**	23	128		20	105	45	3	50	20	10	41	8	5	16	7		2	482	29,59
**may**							8	1	12	2		6	11	10			2	53	3,25
**jun**	3	129	1	14	69	21	1	33	2	19	29	9	46	6			25	407	24,98
**jul**	28	5			90		1	53	5		21	12	37	1			11	264	16,21
**aug**					25					1	3	2	54	18				103	6,32
**sep**					49		12											61	3,74
**oct**					15				3	10	9	3	36					76	4,67
**nov**							5			8	1	8		9	4			35	2,15
**dec**										7		7	26	2	4			46	2,82
**Sum**	54	262	1	34	353	70	33	154	56	57	105	58	215	107	24	5	41	1629	100
**%**	3,31	16,08	0,06	2,09	21,67	4,30	2,03	9,45	3,44	3,50	6,45	3,56	13,20	6,57	1,47	0,31	2,52	100	

In total, 7 crop types were sampled with relatively constant representation across seasons (except for spring-sowed crops in winter). On average, a sector with 10 trapping lines sampled 5.3 annual crop plots (2.24 cereal, 1.24 rape, 0.35 pea, 0.88 maize and sunflower) and 4.7 perennial crop plots, i.e. unploughed for several consecutive years (1.76 alfalfa, 2.32 grassland and 0.97 ray-grass).

The common vole was the main species trapped, accounting for 46% of the catches (3419 males, 2990 females and 1309 individuals of unidentified sex). Living animals were released after being measured. Dead animals were autopsied, recording their weight (Pesola spring balance, 0.5 g precision), sex, body size (total length from nose to tail tip) and number of embryos.

### Vole abundance and individual data

At the trapping line scale, an abundance index was defined as the number of voles caught per 100 trap-nights [31]. At the study area scales, we first averaged trapping indices per crop type then calculated a large-scale index by averaging densities across crop types [26]. We used past June trapping session to calculate an annual index of large-scale density, because this session is constant along the time series and exhibits the highest amplitude of density fluctuation [31].

We only considered individual reproduction parameters in autopsied females (n = 1629). We used two parameters; the presence of embryos (binary variable) and the number of embryos (quantitative variable). It should be noted that embryos are not visually detectable in the first 10 days following fecundation [55] (estimates based on the weight of embryos at birth [56], exponential growth of rodent embryos [59] and the 21 day gestation period in the common vole [56]). The number of pregnant females was thus underestimated but this bias has no reason to vary with density or environmental parameters. The estimated conception date was used to match the date of breeding decision with environmental data, assuming no embryo resorption. In our analyses, we used the estimated conception date, which was 16 days (the median gestation in detectably pregnant females) before the date of trapping. For non breeding females, the environmental data was equally calculated 16 days before trapping.

Body length, even though condition dependent (i.e. dependent to food, season …) in natural environments, may be considered as a proxy of individual age in voles, especially during the first week of life when body growth is rapid and log linear [56]. We calculated body condition [58] score for each animal as the residual from a linear model (Gaussian errors with identity link) of log(body mass) against the body length and the number of embryos (See details in Additional file 1). This latter method was used since carcass mass of females were not available; it assumes that mass of embryo is constant between individuals, which is unlikely but could not be overcome.

### Environmental data

We considered several environmental variables to explain variation in reproduction parameters. Day length was calculated at the centre of the study area (using R package Adehabitat) for each conception date. To differentiate spring and autumn days with similar day lengths but different trajectories, a trend index was also calculated as the change in day length over the past seven days. Food availability was approximated at large scale (i.e. study area scale) by the satellite-derived Normalized Difference Vegetation Index (NDVI), which gives a proxy of plant productivity [59]. We used average values of NDVI for the study area collected by NASA's GIMMS sensor from 1995 to 2006 – pixel resolution at 1 km^2^. As the GIMMS data collection stopped after 2006, we used NDVI data from NASA's MODIS sensor since 2000 to estimate a GIMMS index after 2006 – pixel resolution of 0.0625 square kilometers. We then estimated a value of NDVI for each conception date (details in Additional file 2). Daily temperature data collected at Niort Météo-France station (located within the study area) were used to calculate the average, maximum and minimum temperature at conception date.

**Figure 2:**
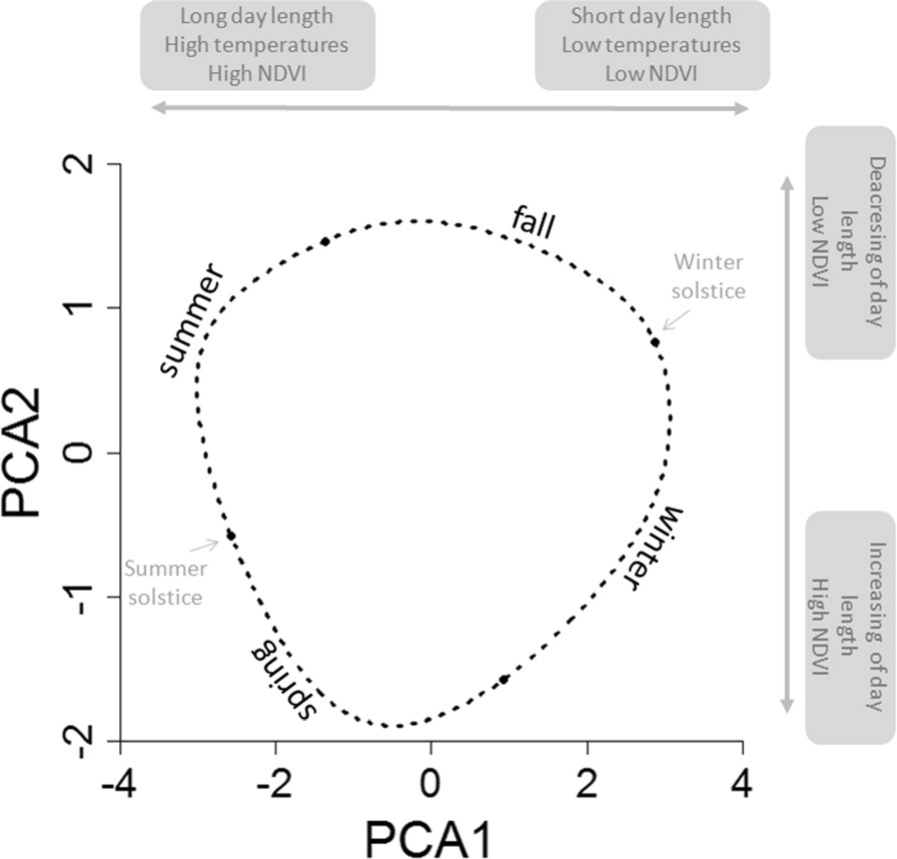
**PCA axis significations.** Schematic representation of the two PCA axes (PCA1 in abscises, PCA2 in ordinates) and how this values of PCA change along the year (dashed line) and across season. For better understanding, the names of seasons and the solstices are indicated.

Environmental data were separated into two distinct categories, representing the intra-annual seasonal variability (average seasonal pattern over multiple years) on the one hand, and inter-annual variability (a proxy of overall season quality) on the other hand. The first set included six environmental variables exhibiting high seasonality (day length, the rate of day length change, NDVI, daily average, maximum and minimum temperatures). The variables were smoothed (with a spline function of the Julian date) using a generalized additive model (package mgcv) to extract their average seasonal pattern across all years (see Additional file 3 for the selection of degree of smoothness). Since the seasonal pattern of the 6 variables were highly correlated (Additional file 3), we used a principal component analysis (PCA) to summarize the information in two uncorrelated axes, summing up 98.9% of the total variance (74.8% component 1, PC1 and 24.1% component 2, PC2; for further details, Additional file 3). The first component mainly explained temperature (minimum, maximum and average daily value), day length, and NDVI. Positive values on this axis represent winter conditions (short day length, cold temperatures and low plant productivity) and are opposed to summer conditions, (Figure 2). The second component predominantly explained day length change and NDVI (Additional file 3). Positives values on the second axis represent fall conditions (day shortening and low plant productivity) whereas negative values represent spring (day length increasing and high plant productivity (see Figure 2). These two principal components were used in the following analyses to resume environmental conditions.

The inter-annual variability ("anomalies with respect to the average values") was only calculated for maximum temperature (that showed more inter-annual variability than minimum or average temperature) and NDVI (average value of the three weeks before the estimated conception date), since patterns of day length are invariable between years. It was calculated as the difference between the variable's actual value and the seasonal average value fitted by the corresponding spline model described above.

### Modelling vole reproduction

To describe variation in breeder fraction and individual reproductive effort (litter size) along the time series, we estimated the linear regression between the fraction of pregnant females and the average number of embryos per litter (excluding zeros, i.e. where female was pregnant) in each trapping session (80 trapping sessions were included in this analysis because 11 of the 91 sessions had no pregnant females and thus litter size could not be estimated). To compare their variance along the year, we used these trapping session averages to calculate a coefficient of variation (standard deviation/mean) for the litter size and the proportion of breeders.

Given the length of our time series, and especially because approximately 60 different people were involved in trapping over the years, reproductive status of captured animal was probably variably recorded. Conversely, data from dead animals were recorded in laboratory, with standardized protocols performed by few technicians. Thus information collected, such as counting the number of embryos, was less subjective. Then, we modelled variation in number of embryos using “hurdle” models [60] to account for an excess of zero counts (n = 1629).

“Hurdle” models are mixture models that include two processes: (1) a binomial component modelling the probability of a count having a zero value, used to determine the factors influencing breeding probability; (2) a truncated Poisson model for the positive litter size counts only (excluding females that did not breed). This allowed to analyze jointly both components of reproductive investment (i.e. engagement and effort) and the variable influencing each component. Variable selection is independent between the two parts of the model.

To select the predictor variables, we used the model with the smallest value of Akaike’ s information criterion (AIC; [61]). We did not use forward or backward stepwise selection procedures because results may be sensitive to the order of inclusion of the candidate variables (e.g. [62,63]). We thus considered all possible models with simple effects and ordered them using AIC (e.g. [63]). Then, relative variables importance (i.e. sum of the AIC weights across all the models in the set where this variable occurred [61]) were calculated in the set of models having a ΔAIC (AIC - AIC of the best model) < 4 in order to compare the relative importance of the different selected variables. At this stage of the procedure, we introduced in the model all candidate variables that, based on literature survey, could be expected to influence common vole reproduction. Specifically, PC1 and PC2 were expected to be correlated with vole reproduction because they summarize the seasonality of environment (food, day length, day length trend, temperature) and those factors are known to influence reproduction in mammals [2,63]. Body length as well as body condition are expected to have positive effects on reproduction [36,37]. Plant productivity and temperature inter-annual variability are equally expected to influence reproduction [34,38,40,47,48]. Finally, we tested the effect of density because density dependence on reproductive parameters may destabilize population dynamics and lead to population cycles [18]. We used trapping line scale as a proxy of current density to test the effect of direct density dependence on reproduction, and past June abundance as a proxy of past density to test the effect of delayed density dependence on reproduction. We tested the effect of direct and delayed density dependence on reproduction based on supposed effects on cyclic dynamics [13,16].

Given the high number of explanatory variables, model selection was performed following two steps. First, all explanatory variables were tested without accounting for interactions to select those relevant to explain reproduction all over year. Hence all combinations of the 8 variables (PC1, PC2, NDVI anomalies, temperature anomalies, body condition, body length, current abundance and past June abundance) were tested in both part of the “hurdle” model (count and binomial) from null model (intercept only) to full model (one coefficient estimated for each variables in both parts of the model). Seasonal changes (i.e. PCA axes) were added as main effects because reproduction of common vole is known to be seasonal [35,42]. Taking account of that seasonality permits to better estimate coefficients like intercept. Furthermore, we used PCA axes in a second part of the analyses in interaction with other variables to test seasonality dependence of other variables. To be sure to select, during the selection process, an interaction (e.g. density * seasonality) and not the information added by the PCA axis, we had to add the PCA axes in main effect in addition to the interaction. In a second step, to investigate the expected seasonal dependant influence of the variables accounting for reproduction, the interactions between the selected variables in the first step of model selection and seasonal variables (i.e. PCA axes) were tested. Interaction terms always included the seasonal variable to account for possible changes in the influence of variables with seasons. For ease of interpretation, only the 25 best models are presented (Additional file 4). Non-linear regressions may have fitted data more accurately, however we preferred simple conventional linear models to avoid complexity or overfitting.

All statistical analyses were performed using R software (R Project Core Development Team, Vienna, Austria [65]) version 2.11. We fit extended hurdle models with the pscl library [66,67] and did model averaging with MuMin library.

## Results

### Seasonal pattern in female productivity

The average presence and number of embryos showed a similar pattern of seasonal variation with higher values in spring and summer. The two seasonal patterns were positively correlated (coefficient of regression and 95% confidence interval: 0.13 [0.086; 0.175], n = 80). The coefficients of variation among seasons was higher in the probability of being pregnant than in the number of embryos (0.497 and 0.186 respectively).

### Model selection

#### Simple effect models

**Figure 3:**
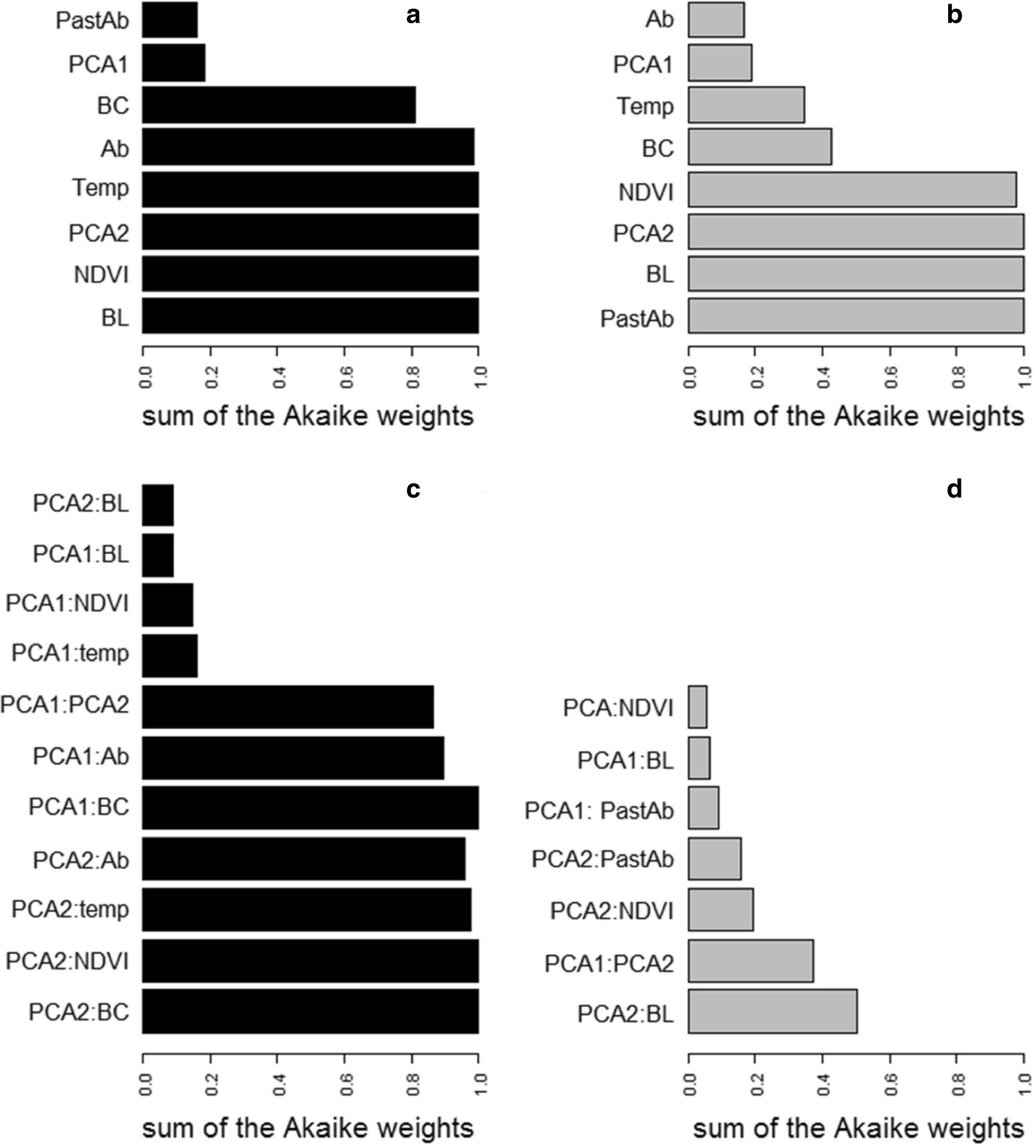
**Variable weight in models with ΔAIC < 4.** Results of variable weight on the set of model with ΔAIC < 4, performed on the first step of model selection (**(a)** and **(b)**) and on the second step of model selection (**(c)** and **(d)**). Variable weights of the breeding probability part of the model are in black (**(a)** and **(c)**) and variable weights of the litter size part of the model in grey (**(b)** and **(d)**). (variable definition; PastAb: past June abundance, PCA1: first axe of PCA, BC: body condition, Ab: current abundance, Temp: anomalies of temperatures, PCA2: second axe of PCA, NDVI: anomalies of NDVI, BL: body length)

Among the 65536 models without interaction, 36 had a ΔAIC < 4. Body length, NDVI anomalies, PC2, Temperature anomaly, current abundance and body condition were selected with a variable AIC_w_ ≥ 0.5 in the binomial part (i.e. breeding probability) (Figure 3a). On the other hand, past June abundance, body length, PC2 and NDVI anomalies were included with a variable AIC_w_ ≥ 0.5 in the count part (i.e. litter size) of the set of models with ΔAIC < 4 (Figure 3b). These two sets of variables were then used in the second step to run models with interaction (Additional file 4).

#### Interacting effect models

Among the 282880 models including interactive terms, 117 had ΔAIC < 4. According to the weight of variable esteemed from the set of models with ΔAIC < 4 (Figure 3c and d), interactions including seasonal variables were consistently selected. Six of these were involved in explaining breeding probability: PC2 with body condition, NDVI anomalies, temperature anomalies, current abundance; and PC1 with body condition, current abundance and PC2 (Figure 3c). Conversely only one interaction obtained a variable AIC_w_ ≥ 0.5 in the litter size part of the models; the interaction between PC2 and body length (Figure 3d).

Because it appeared to be the most parsimonious model, we chose to analyze the model with variables obtaining highest AIC_w_ ≥ 0.5). That model obtained also the smallest AIC score (Additional file 4). This *best* “hurdle” model explained 14.43% of the total deviance, and included an intercept, 7 main effects and 7 interactions in predicting the probability of being pregnant and an intercept, 4 main effects and 1 interaction in predicting the litter size (Table 3).

**Table 3:** Best model parameters

**ZERO PART of the best model (explaining breeding probability)**				
**Explanatory variable**	**Estimate**	**Std. Error**	**z value**	**p-value**
(Intercept)	-6.0575	0.4876	-12.4242	0.0000
PCA1	0.0568	0.0594	0.9567	0.3387
PCA2	-0.2772	0.0972	-2.8509	0.0044
BL	0.0495	0.0041	11.9786	0.0000
BC	0.7641	0.4746	1.6100	0.1074
NDVI	0.0123	0.0032	3.7885	0.0002
Temp	-0.0017	0.0291	-0.0595	0.9526
Ab	-0.042	0.0132	-3.1861	0.0014
PCA2 : BC	0.9505	0.3198	2.9722	0.003
PCA2 : NDVI	-0.0074	0.0027	-2.738	0.0062
PCA2 : Temp	0.0505	0.0232	2.1708	0.0299
PCA2 : Ab	-0.0202	0.0078	-2.5672	0.0103
PCA1 : BC	0.5083	0.1905	2.6685	0.0076
PCA1 : Ab	-0.0094	0.0045	-2.0955	0.0361
PCA1 : PCA2	-0.0904	0.0480	-1.8814	0.0599
**COUNT PART of the best model (explaining litter size)**				
**Explanatory variable**	**Estimate**	**Std. Error**	**z value**	**p-value**
(Intercept)	0.578	0.2407	2.4013	0.0163
PCA2	-0.3396	0.1659	-2.0466	0.0407
BL	0.0082	0.0019	4.3701	0.0000
NDVI	0.0022	0.0010	2.2656	0.0235
PastAb	-0.0086	0.0043	-2.022	0.0432
PCA2 : BL	0.002	0.0013	1.5126	0.1304

Best model parameters explaining common vole female per capita productivity (number of embryos per females) in Western France. Model used was mixture model (“hurdle”) with two parts: a zero part explaining the breeding probability with binomial distribution and logit link (zero versus superior to zero); the second part explaining the litter size (distribution of values superior to zero occulting all zero counts) with a Poisson distribution and a log link (variable definition; PastAb: past June abundance, PCA1: first axe of PCA, BC: body condition, Ab: current density, Temp: anomalies of temperatures, PCA2: second axe of PCA, NDVI : anomalies of NDVI, BL: body length).

### Environmental variability – seasonal trends

**Figure 4:**
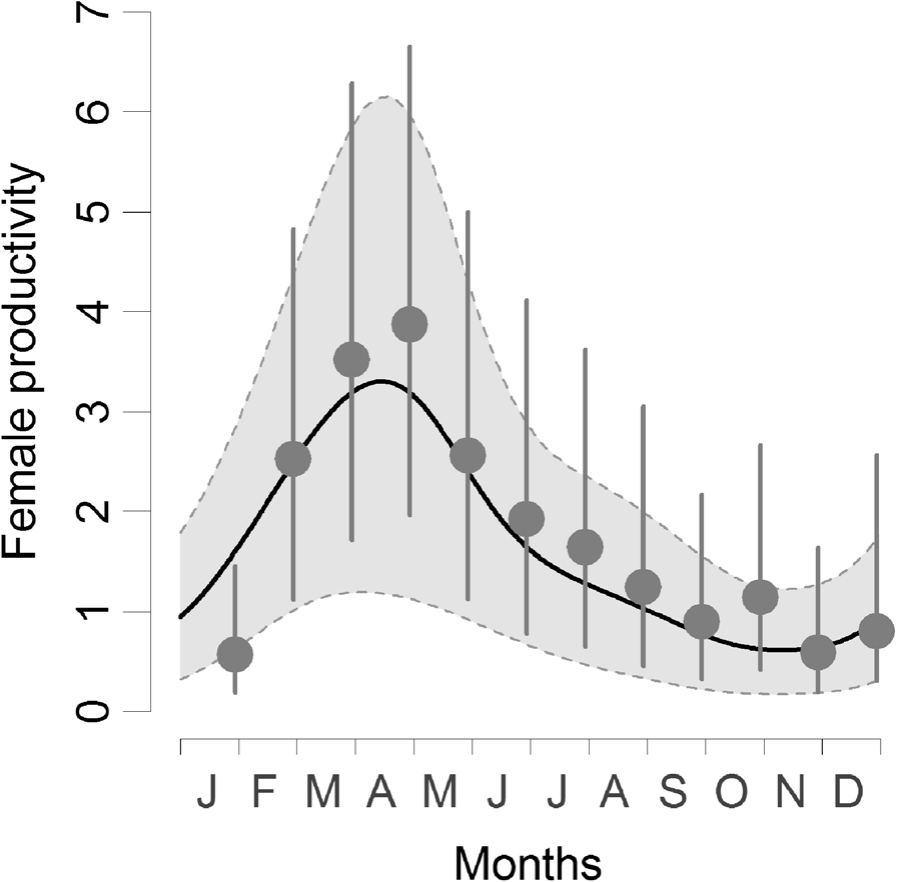
**Effect of intra-annual environmental seasonality on reproduction.** Seasonal pattern of vole female per capita productivity (number of embryos per females) along year. The monthly average per capita embryo production (circles) with 80 percent confidence interval is calculated from field data. For the prediction (solid line with shaded area indicating the 70% confidence interval of coefficients estimate), only the seasonal component of the environment (the two components from the PCA summarizing 98.9% of the environment seasonality) was allowed to vary with the date. All other explanatory variables (size, body condition, abundances, plant productivity anomalies) were fixed at their mean value. Also the seasonal pattern of common vole reproduction is well explained by the seasonal variation of environment.

PC1 only affected breeding probability while PC2 affected negatively both litter size and breeding probability. Furthermore, an interaction between PC1 and PC2 was selected in the breeding probability part of the model. Those results indicated that among seasonal variables, day length trend and NDVI predominantly influenced the seasonal pattern of female common vole reproduction. Voles bred and invested more in reproduction when day length increased and when NDVI were high. PC axes therefore explained well the seasonal pattern of common vole reproduction (see Figure 4).

### Environmental variability – deviations from seasonal trends

**Figure 5:**
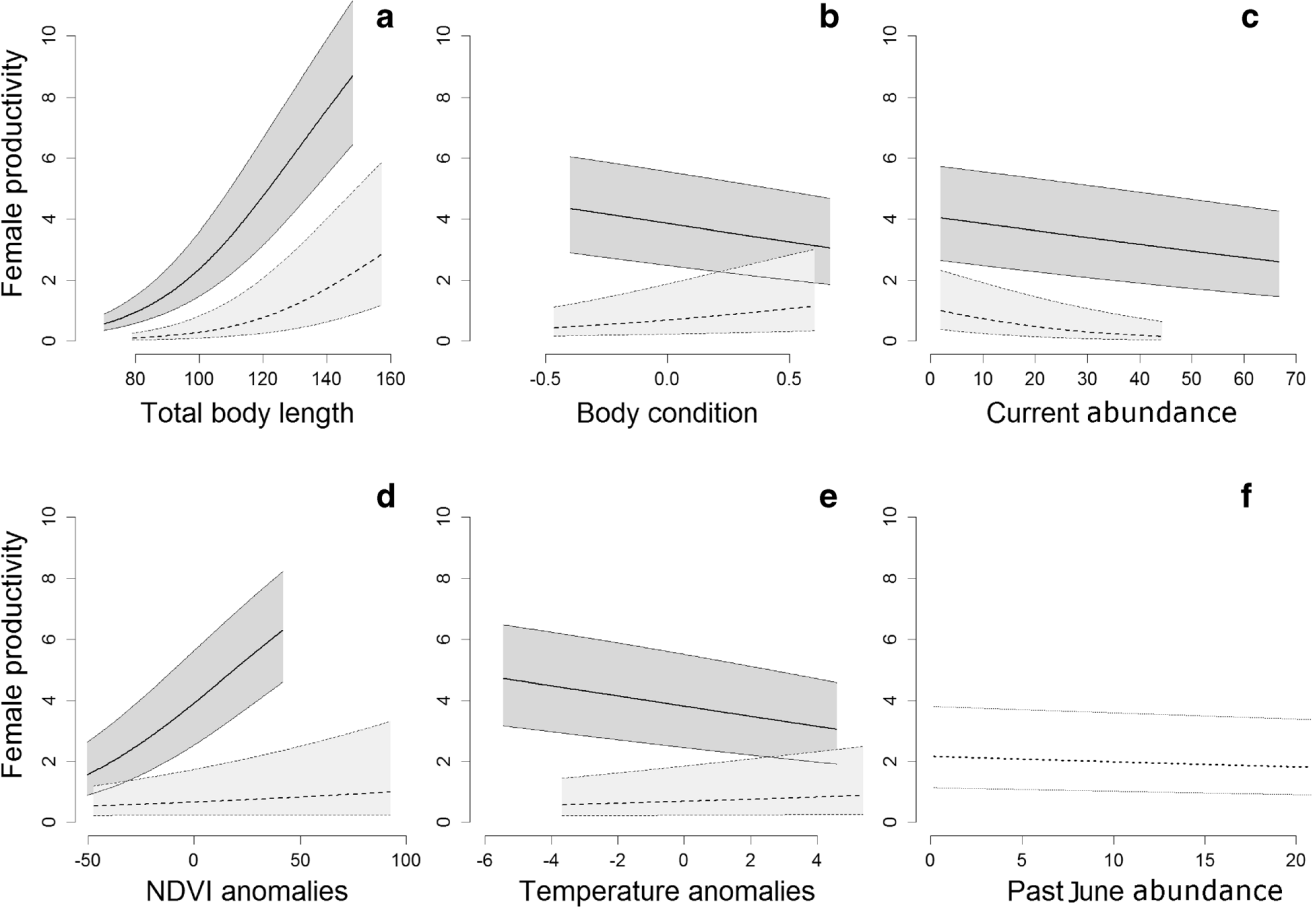
**Effect of intrinsic and extrinsic parameters on reproduction.** Average vole female per capita productivity (average number of embryos per females) in relation to **(a)** body length, **(b)** body condition, **(c)** current local abundance, **(d)** NDVI anomalies, **(e)** temperatures anomalies and **(f)** past June abundances. For models including interaction with seasonal component of environment, the relation between the female production and the explanatory variable was represented in dark grey for spring (16th February-15th May), and in light grey for the fall (16th August-15th November). For each panel, only the variable on the X-axis changes; the other variables are held constant at their mean value for the season. Fifty percent coefficient confidence intervals are drawn.

NDVI anomalies affected both breeding probability and litter size positively, but less so in fall than in winter for the breeding probability as suggested by the interaction between NDVI and PCA2. Figure 5d shows the influence of NDVI anomalies on female productivity (i.e. breeding probability * litter size).Temperature anomalies only affected the breeding probability; positively in fall and winter and negatively in spring and summer as suggested by the interaction with PCA2 (Figure 5e).

### Individual morphological characteristics

Body length affected both breeding probability and litter size positively, but less so in spring and summer for the litter size as suggested by the interaction between body length and PCA2. Figure 5a shows the influence body length on breeding probability in spring and fall.Body condition only affected the breeding probability. Its influence was positive in winter (higher) and fall (less high) and negative in summer (higher) and spring (less high) as suggested by the interactions between body condition and PCA1 and body condition and PCA2 (Figure 5b).

### Effects of vole density

Current vole abundance had a negative effect on breeding probability. However, this effect was stronger in fall and winter as suggested by interactions with PCA1 and PCA2. The effect of local density was thus dependent on season, showing a near-complete halt in reproduction in fall and winter when outbreak occurs. The negative effect of density was maximal in winter (predicted loss of 86% of the average number of embryos per female from observed values), fall (85%), spring (35%) and summer (14% - Figure 5c for spring and fall representation).The past June abundance affected litter size. The effect was constant during seasons (no interactions with seasonal variables selected - Figure 5f).

## Discussion

The two components of vole breeding effort (litter size and breeding probability) had a similar seasonal pattern. The fraction of breeders showed a higher variability than litter size, suggesting that the main part of variance in productivity is due to the probability of breeding. All variables (except past June abundance) showed a significant interaction (although sometimes of a small magnitude) with seasonal trends, highlighting that the effects of environment (NDVI and temperature anomalies), morphology (body length and body condition) and density on reproduction, all vary seasonally. Density had a negative effect on reproduction at the end of the breeding season, with negligible reproductive activity when field-level autumn density exceeded an index of 15 (outbreak abundance, Figure 5c).

In this study, the number of pregnant females was probably underestimated since we cannot detect embryos in the first half of the 21 days of pregnancy. Therefore our index was probably relevant. It probably helps explaining, in addition to complex structure of the explained variable, why the best model explained only 14.43% of total deviance.

### Vole reproduction in a seasonal environment

Common vole reproduction is characterized by a strong seasonal pattern. This pattern is well explained by the PCA summarising seasonal patterns in day length, day-length trends, NDVI and temperatures (Figure 4). The relative contribution of each variable to this pattern cannot be ascertained due to colinearity between variables. However, this is in agreement with previous experimental studies on common vole [46,47] suggesting that breeding phenology is controlled by day length and food quality. Moreover, we showed that NDVI anomalies have a large impact on both the breeding probability and litter size, though only during the main breeding season (spring and summer), which can be as unproductive as autumn and winter when the plant productivity anomaly is very low (Figure 5d). This pattern is particularly clear, despite the low spatial precision of the NDVI data used (averaged over the study area) relative to a common vole's home range (a few hundreds of square meters [56,57]). NDVI is thus probably a good proxy of resources available to common voles at a large scale. The large impact of NDVI anomalies in spring and summer suggests that plant productivity is a major limiting factor of common vole reproduction, consistent with other studies [18,35,43]. Furthermore, the reproduction decrease starting from the end of summer regardless of NDVI anomalies and temperature anomalies, suggests that the decrease of day length constrains reproduction during autumn. Short day length has been shown to inhibit reproduction [44,45,47] and may be the main limiting factor impacting reproductive rate during winter. Seasonality of rodent induced by seasonality of environment is a quasi compulsory adaptation in temperate and Nordic latitudes to match juvenile growth to food availability [2]. In several studies involving rodents, an addition of food resulted in an increase of reproduction [7-12]. However, it is less clear whereas natural inter-annual variation of food availability is able to influence reproduction to a similar extent. Nevertheless, vegetation productivity varies also under the influence of temperature and precipitation, suggesting that large climatic fluctuations may impact common vole population dynamics (see also [68]). We found that the inter-annual variability of plant productivity could have strong effect on reproduction, particularly in spring and summer, and thus possibly on population dynamics. A possible example may be the year 2007, when major common vole outbreaks occurred in several European countries [28], including this area, where an outbreak was not expected until 2008 (due to 3-years cyclicity). This outbreak occurred after a 9 months long, strong positive anomaly of plant productivity (more than four times greater than other anomalies between 1981 and 2010) observed all over Europe, suggesting a Moran effect (see [69] for theory and [70] for an empirical demonstration), synchronising common vole outbreaks across Europe. Low plant productivity may prevent high reproductive rates during the main breeding season. This implies that common voles could not reach high density in a non productive environment and means that high plant productivity may be a prerequisite for common vole population cycles. In several cases, population cycles were linked to climate variability (especially North Atlantic Oscillation, e.g. in Europe [70] and in North America [71,72]) and direct link between food availability and oscillations was suggested [70] even if food is described more as a limiting factor impacting winter survival than a limiting factor impacting spring population growth rate.

### Morphological characteristics influencing reproduction

Individual morphological characteristics are strong drivers of both the individual likelihood of being pregnant and litter size. As expected, larger females are more likely to be pregnant and have larger litters (Figure 5a). These results suggest that the effect of body size on reproduction results from the ability of female common voles to reproduce from the age of 2 weeks in extreme cases [37,73], long before reaching their “longest” body size (after 6 months of age; [36,56]). Young (i.e. small) females reproduce less and produce smaller litters than older ones due to allometric constraints. However, the relation between body size and reproductive status is variable within years (Figure 5a). Our results suggest, consistently to others studies (e.g., [74]), that voles may breed more precociously (i.e. at a smaller size) during spring and summer (see also [42]) while bigger females may breed throughout the year (albeit at a reduced rate in autumn and winter). The higher relative cost of reproduction during fall and winter probably (less resources, binding climatic conditions) explains why smaller females in fall and winter have to delay their reproduction. Those effects are well known in small rodent [e.g. [37,74,75]. However, the variation of reproduction cost along year is partially due to environmental seasonality (temperature, day length, food availability: [2,64]) and therefore vole cohorts may differ with regard to body size, since the latter is generated by adaptation to environmental seasonality. The effect of the seasonality of body size on reproduction is thus partially induced by environmental seasonality.

Body condition appears to be an important determinant of breeding status and shows a clear seasonal pattern, with negative effects on productivity during spring and summer, but positive effects in autumn and winter (Figure 5b). The positive influence of body condition on reproduction during autumn and winter suggests that only the fattest females are able to reproduce during the less favourable seasons and that they remain in better condition than others, a rather general result [58]. In contrast, the negative association between body condition and reproduction in spring and summer suggests that females lose weight during pregnancy in these seasons. This latter result is more difficult to explain since food availability is higher in that period. It could be a spurious relationship due to the fact that female breeding status is biased due to our measurement method (pregnancy is not detectable before 10 days), leading to false zero represented by undetected reproductive females.

### Density dependence

Density is known to greatly vary in space and time in western France common vole population (Figure 1a, [76,77]). However, density does not appear to have a strong impact on the proportion of breeding females or on litter size during the core breeding season (see also [26]) but we found evidence that the proportion of breeders is reduced during the high density phase (Figure 5c). Due to the relation between body size and reproduction, during autumn and winter, only the bigger common voles are able to reproduce, but they reproduce less. This reduction of reproduction rate in autumn and winter becomes stronger at high population density, up to a complete halt in breeding at the highest local densities (Figure 5c). Additionally, individuals are smaller after a population crash [26] and smaller individuals produce smaller litters [35]. An additive negative effect of past June abundance was found on litter size, resulting in a litter size reduction of 28% in springs following crashes. A possible explanation is that the peak induces a reduction of individual quality through developmental disorders or maternal effects [78]. This may result in a decrease in the population growth rate the year following the peak and contribute to a prolonged low phase *sensu* Boonstra et al. [79].

Despite the observation of density dependence, population regulation is fundamentally different in this system compared to *M. agrestis* (the closest “cyclic” species) in northern England [21,22]. In the latter system, one-year delayed density-dependence affected the length of the breeding season contributing to a crash due to an absence of recruitment. In our case, the length of the breeding season may be decreased by an early halt to reproduction at high current densities which is compatible with explaining crashes by an absence of recruitment including its timing [22,23]. However, contrary to *M. agrestis* in northern England, only direct density dependence was observed before crash and thus delayed density dependence cannot induce the decline in the studied common vole population.

## Conclusions

Environmental seasonality variables (day-length, temperature, plant productivity) were the main drivers of common vole reproduction in central-western France. Among environmental variables, plant productivity appeared to have a critical role, determining inter-annual variability of female common vole reproduction. Hence, the peak of vole reproduction occurred in spring, when environment is highly productive. This suggests that outbreak abundances (i.e. several thousands per hectare) can be only reached in areas where plant productivity is high during several months per year, which is consistent with the absence of record, to our knowledge, of common vole cyclic dynamics in non agricultural landscape (for a French population in non agricultural landscape, see [80]). Modern agriculture, with the enhancement of plant productivity may have promoted common vole outbreaks by favouring their reproduction.

During the less favourable seasons, intrinsic factors and particularly local density strongly impacted common vole reproduction. Given the low survival of common vole (a few months), a stop of reproduction may cause a drastic population decline in a short time. In the case of western France common vole cycles, this suggests that the decline phase following outbreaks may have been partly or totally caused by direct density dependence, conversely to the declines observed in northern rodent cycles. Furthermore, this study demonstrates that researchers should spend more time on measuring vole reproduction outside of the main activity season.

## Competing interests

The authors declare that they have no competing interests.

## Authors’ contributions

VB designed and is responsible of the data time series. Many people performed the field and lab work from 1995 to 2011, including VB, BG, AP. AP, VB, BG designed the study. AP analyzed the data. AP, BG, VB wrote the manuscript. All authors read and approved the final manuscript.

## Supplementary Material

Additional file 1 Calculation of the body condition index. Click here for file

Additional file 2 Compilation of plant productivity time series. Click here for file

Additional file 3 Summarizing environmental seasonality. Click here for file

Additional file 4 25 best models of AIC selection. Click here for file
