# Supplementary material for: The interplay between seasonality and density: consequences for female breeding decisions in a small cyclic herbivore
Source: BMC Ecol. 2014 May 28;14:17. doi: 10.1186/1472-6785-14-17 (PMC4049426; doi:10.1186/1472-6785-14-17)
Supplement: Additional file 1 — Calculation of the body condition index. [file 1472-6785-14-17-S1.docx]

**Additional file 1 – Calculation of the body condition index**

Model details and parameters estimations for calculus of body condition index.

Explained data: log (weight)

Explanatory variables: body length, number of embryos

Model used: Gaussian generalized linear model with identity link

The model explained 71.7 % of the total deviance.

Table 1 summarise the best model.

**Table 1: Best model for body condition index estimation.**

|  | explained deviance | % of total deviance explained | estimate | SE | t value | p-value |
| --- | --- | --- | --- | --- | --- | --- |
| intercept |  |  | 0.8482736 | 0.0372621 | 22.77 | <2e-16 |
| Body length | 141.597 | 63.7 | 0.0165869 | 0.0003278 | 50.60 | <2e-16 |
| Number of embryos | 16.744 | 8.0 | 0.0385762 | 0.0018128 | 21.28 | <2e-16 |
