# Supplementary material for: The interplay between seasonality and density: consequences for female breeding decisions in a small cyclic herbivore
Source: BMC Ecol. 2014 May 28;14:17. doi: 10.1186/1472-6785-14-17 (PMC4049426; doi:10.1186/1472-6785-14-17)
Supplement: Additional file 2 — Compilation of plant productivity time series. [file 1472-6785-14-17-S2.docx]

**Additional file 2 – Compilation of plant productivity time series**

Aim; Compilation of two NDVI time series collected from different sensors.

We used 22-day average values of NDVI for the study area collected by NASA's GIMMS sensor from 1995 to 2006. As the GIMMS data ended in early 2007, we used NDVI data from NASA's MODIS sensor (recorded every 16 days since 2000) to estimate a GIMMS index after 2007. The six full years of overlap were used to calibrate the two NDVI estimators. There was a high correlation between the two time series (fig. 1) providing a satisfactory relationship between GIMMS and NDVI values (using corresponding value of GIMMS for each NDVI data. every 16 days; N=154. slope=0.056. p<0.0001. R² = 0.59)

A Gaussian generalized linear model with identity link was used to predict NDVI recorded by the GIMMS sensor from the NDVI recorded by the MODIS sensor as well as several climatic variables (day length, Julian date from 1 January, Météo France : Average temperature, maximum daily temperature, minimum daily temperature, snow cover). The summary of the best model is available in the table 2. The best model explained 78.24% of the total deviance.

We controlled the ability of the best model to predict GIMMS NDVI values from MODIS NDVI data and others climatic variables by testing the correlation between GIMMS NDVI values and estimated GIMMS NDVI values during the period of overlap (fig. 2). The R-squared of the relation was 0.78.

Then we obtained a time series for NDVI from 1985 to 2010 (fig. 3)

**Table 2:** **Selected model for predictions of GIMMS NDVI values by MODIS NDVI and various climatic variables.**

|  | explained deviance | % of total deviance explained |  |
| --- | --- | --- | --- |
| MODIS NDVI daily average | 3550473.698 | 46.92 | |
| MODIS NDVI daily anomaly* | 1677448.846 | 22.17 | |
| Average temperature | 107448.4732 | 1.42 | |
| Maximum daily temperature | 88960.94417 | 1.18 | |
| Minimum daily temperature | 22164.59464 | 0.29 | |
| Snow cover | 3653.894782 | 0.05 | |
| Day length | 450490.7922 | 5.95 | |
| Julian date from each 1 January | 15577.21554 | 0.21 | |

* Deviation from average value (see “appendix 3; seasonal pattern of environment” for estimation of average value)


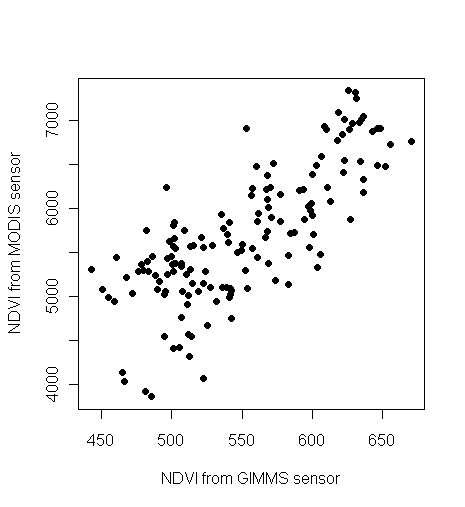


**Fig. 1 Correlation between NDVI data estimated from two different sensors during the same period.** Correlation of NDVI measurements collected every 22 days from the GIMMS sensors and the nearest temporal value of NDVI collected by MODIS sensor from 2000 to 2006.

**Fig. 2** **Correlation between NDVI from original GIMMS data and NDVI predicted from MODIS data.**

**
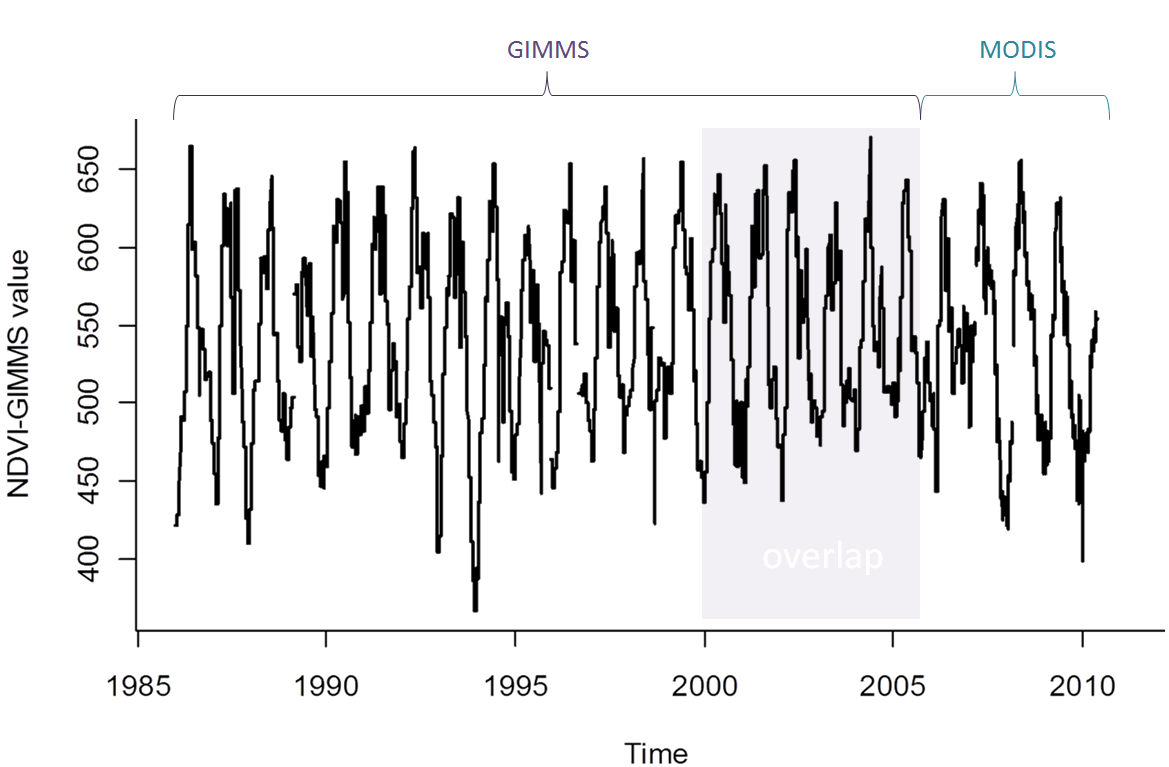
**

**Fig. 3** **Reconstructed NDVI time series used for the analysis**
