# Supplementary material for: The interplay between seasonality and density: consequences for female breeding decisions in a small cyclic herbivore
Source: BMC Ecol. 2014 May 28;14:17. doi: 10.1186/1472-6785-14-17 (PMC4049426; doi:10.1186/1472-6785-14-17)
Supplement: Additional file 3 — Summarizing environmental seasonality. [file 1472-6785-14-17-S3.docx]

**Additional file 3 - Summarizing environmental seasonality**

Aim: compilation of several correlated environmental variables for obtaining uncorrelated indices characterising the environmental seasonality.

Environmental variable:

- day length
- daily day length change
- daily maximum temperature
- daily minimum temperature
- daily average temperature
- NDVI

**Seasonal pattern of environment:**

For all seasonal variable exhibiting variability between year (daily min, max and average temperatures and NDVI) we estimated the average seasonal pattern by smoothing the Julian day from the first January of each year with generalized additive model (GAM, table 3). Number of equivalent degrees of freedom (edf) in the spline function was selected by Newton type optimization of AIC [1]; Data time series from 1986 to 2010 were used.

**Summary of environmental seasonal pattern**

As all variables follow high correlated seasonal pattern (table 4), we summarised environmental seasonality with principal component analysis. We used daily estimated average value (from GAM) for NDVI daily min, max and average temperatures and real values of day length and day length trends (calculated as the change in day length over the past seven days). Also the number of sample was equal to 365.

Table 5 and figure 4 and 5 summarise the result of ACP: Figure 4 gives the eigenvalues for axes. Figure 5 summarises the origin of information supported by the 2 first axes (as well as table 5) and shows how the values of this two axes evolve throughout the seasons.

**Table 3:** **Models summary**

| **Explanatory variable** | **explained deviance** |  | **value** | **se** | **p-value** |
| --- | --- | --- | --- | --- | --- |
| NDVI | 70,50% | intercept | 540,1027 | 0,35 | < 1e-6 |
|  |  | edf | 7,795 |  | < 1e-6 |
|  |  |  |  |  |  |
| Av temp | 72,40% | intercept | 12,13619 | 0,03446 | < 1e-6 |
|  |  | edf | 7,417 |  | < 1e-6 |
|  |  |  |  |  |  |
| Min temp | 73,10% | intercept | 17,27857 | 0,04032 | < 1e-6 |
|  |  | edf | 6,879 |  | < 1e-6 |
|  |  |  |  |  |  |
| Max temp | 61,40% | intercept | 7,56102 | 0,03668 | < 1e-6 |
|  |  | edf | 7,649 |  | < 1e-6 |

**Table 4** Correlation between variables in the principal components analysis (based on daily estimation)

|  | Day length seasonal pattern | Daylength trends seasonal pattern | NDVI seasonal pattern | Av. Temperature seasonal pattern | Max. Temperature seasonal pattern | Min. Temperature seasonal pattern |
| --- | --- | --- | --- | --- | --- | --- |
| Day length seasonal pattern | / | 0 | 0.92 | 0.86 | 0.88 | 0.81 |
| Daylength trends seasonal pattern |  | / | 0.29 | 0.5 | 0.46 | 0.57 |
| NDVI seasonal pattern |  |  | / | 0.63 | 0.67 | 0.56 |
| Av. Temperature seasonal pattern |  |  |  | / | 1 | 1 |
| Max. temperature seasonal pattern |  |  |  |  | / | 0.95 |
| Min. temperature seasonal pattern |  |  |  |  |  | / |

**Table 5** Composition of the first two principal components.

|  | Component 1 | Component 2 |
| --- | --- | --- |
| Day length | -0.92 | -0.38 |
| Daylength trends | 0.37 | -0.92 |
| Plant productivity | -0.75 | -0.64 |
| Av. Temperature | -0.99 | 0.15 |
| Max. temperature | -0.99 | 0.11 |
| Min. temperature | -0.97 | 0.24 |


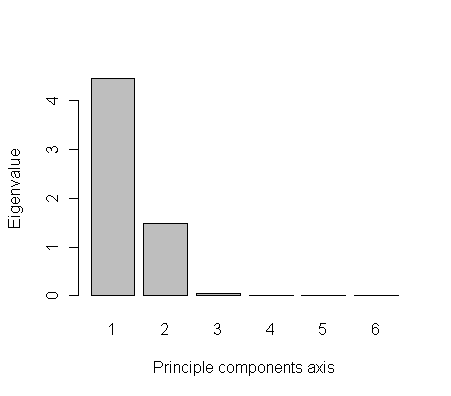


**Fig. 4** **Eigenvalues for each axis of the principal components analysis.**


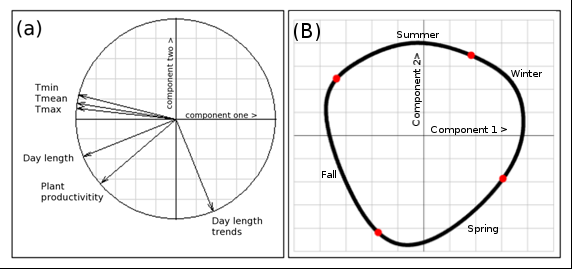


**Fig. 5 Summarise of PCA.** a) - Correlation circle of the PCA. The direction and the length of each arrow shows the contribution of the corresponding variable to each of the first two components as well as illustrating the correlations among variables. b) – Seasonal variation in the first two principal components during the year. The red dots are the threshold points between seasons.
