# Supplementary material for: The interplay between seasonality and density: consequences for female breeding decisions in a small cyclic herbivore
Source: BMC Ecol. 2014 May 28;14:17. doi: 10.1186/1472-6785-14-17 (PMC4049426; doi:10.1186/1472-6785-14-17)
Supplement: Additional file 4 — 25 best models of AIC selection. [file 1472-6785-14-17-S4.docx]

**Additional file** **4 – 25 best models of AIC selection**

**Variable definition**; PastAB : past June abundance, PCA1 : first axe of PCA, BC : body condition, Ab : current density, Temp : anomalies of temperatures, PCA2 : second axe of PCA, NDVI : anomalies of NDVI, BL : body length

25 best models for procedure selection without interactions:

|  | **Count part (litter size)** | **Binomial part (breeding proba)** | **AIC** | **loglik** | **npar** | **deltaAIC** |
| --- | --- | --- | --- | --- | --- | --- |
| 1 | PCA2 + BL + NDVI + Past Ab | PCA2 + BL + BC + NDVI + Temp + Ab | 4425.17587475872 | -2200.58793737936 | 12 | 0 |
| 2 | PCA2 + BL + NDVI + Temp + Past Ab | PCA2 + BL + BC + NDVI + Temp + Ab | 4425.82512052702 | -2199.91256026351 | 13 | 0.649245768298897 |
| 3 | PCA2 + BL + BC + NDVI + Past Ab | PCA2 + BL + BC + NDVI + Temp + Ab | 4426.43151841009 | -2200.21575920505 | 13 | 1.25564365137325 |
| 4 | PCA2 + BL + BC + NDVI + Past Ab | PCA2 + BL + NDVI + Temp + Ab | 4426.63951008198 | -2201.31975504099 | 12 | 1.46363532325813 |
| 5 | PCA1 + PCA2 + BL + NDVI + Past Ab | PCA2 + BL + BC + NDVI + Temp + Ab | 4427.01170470332 | -2200.50585235166 | 13 | 1.8358299445963 |
| 6 | PCA2 + BL + NDVI + Past Ab | PCA1 + PCA2 + BL + BC + NDVI + Temp + Ab | 4427.0274103247 | -2200.51370516235 | 13 | 1.85153556597561 |
| 7 | PCA2 + BL + BC + NDVI + Temp + Past Ab | PCA2 + BL + BC + NDVI + Temp + Ab | 4427.08674504839 | -2199.54337252419 | 14 | 1.91087028966831 |
| 8 | PCA2 + BL + NDVI + Ab + Past Ab | PCA2 + BL + BC + NDVI + Temp + Ab | 4427.09060176433 | -2200.54530088216 | 13 | 1.91472700560735 |
| 9 | PCA2 + BL + NDVI + Past Ab | PCA2 + BL + BC + NDVI + Temp + Ab + Past Ab | 4427.17445826181 | -2200.58722913091 | 13 | 1.99858350308932 |
| 10 | PCA2 + BL + BC + NDVI + Temp + Past Ab | PCA2 + BL + NDVI + Temp + Ab | 4427.29473672027 | -2200.64736836014 | 13 | 2.11886196155319 |
| 11 | PCA1 + PCA2 + BL + NDVI + Temp + Past Ab | PCA2 + BL + BC + NDVI + Temp + Ab | 4427.52115712448 | -2199.76057856224 | 14 | 2.34528236575534 |
| 12 | PCA2 + BL + NDVI + Temp + Past Ab | PCA1 + PCA2 + BL + BC + NDVI + Temp + Ab | 4427.676656093 | -2199.8383280465 | 14 | 2.50078133427451 |
| 13 | PCA2 + BL + NDVI + Temp + Ab + Past Ab | PCA2 + BL + BC + NDVI + Temp + Ab | 4427.79830110072 | -2199.89915055036 | 14 | 2.62242634200356 |
| 14 | PCA2 + BL + NDVI + Temp + Past Ab | PCA2 + BL + BC + NDVI + Temp + Ab + Past Ab | 4427.82370403011 | -2199.91185201505 | 14 | 2.64782927138731 |
| 15 | PCA1 + PCA2 + BL + BC + NDVI + Past Ab | PCA2 + BL + BC + NDVI + Temp + Ab | 4428.27993935767 | -2200.13996967883 | 14 | 3.10406459894875 |
| 16 | PCA2 + BL + BC + NDVI + Ab + Past Ab | PCA2 + BL + BC + NDVI + Temp + Ab | 4428.2801514005 | -2200.14007570025 | 14 | 3.10427664177496 |
| 17 | PCA2 + BL + BC + NDVI + Past Ab | PCA1 + PCA2 + BL + BC + NDVI + Temp + Ab | 4428.28305397607 | -2200.14152698803 | 14 | 3.10717921734795 |
| 18 | PCA2 + BL + Past Ab | PCA2 + BL + BC + NDVI + Temp + Ab | 4428.30100707533 | -2203.15050353766 | 11 | 3.12513231660887 |
| 19 | PCA2 + BL + BC + NDVI + Past Ab | PCA2 + BL + BC + NDVI + Temp + Ab + Past Ab | 4428.43010191318 | -2200.21505095659 | 14 | 3.25422715446075 |
| 20 | PCA2 + BL + BC + NDVI + Past Ab | PCA1 + PCA2 + BL + NDVI + Temp + Ab | 4428.47587720227 | -2201.23793860113 | 13 | 3.30000244354505 |
| 21 | PCA1 + PCA2 + BL + BC + NDVI + Past Ab | PCA2 + BL + NDVI + Temp + Ab | 4428.48793102955 | -2201.24396551478 | 13 | 3.31205627083364 |
| 22 | PCA2 + BL + BC + NDVI + Ab + Past Ab | PCA2 + BL + NDVI + Temp + Ab | 4428.48814307238 | -2201.24407153619 | 13 | 3.31226831365984 |
| 23 | PCA2 + BL + BC + NDVI + Past Ab | PCA2 + BL + NDVI + Temp + Ab + Past Ab | 4428.61649560519 | -2201.3082478026 | 13 | 3.44062084646976 |
| 24 | PCA1 + PCA2 + BL + BC + NDVI + Temp + Past Ab | PCA2 + BL + BC + NDVI + Temp + Ab | 4428.79908586396 | -2199.39954293198 | 15 | 3.6232111052359 |
| 25 | PCA1 + PCA2 + BL + NDVI + Past Ab | PCA1 + PCA2 + BL + BC + NDVI + Temp + Ab | 4428.86324026929 | -2200.43162013465 | 14 | 3.68736551057191 |

25 best models for procedure selection with interactions:

Only the interactions are presented in that table, those interactions were added to the best model without interactions (first model of the previous table). *Note that when PCA1 appears in the interaction, the single term (PCA1) was also added*.

|  | **Count part (litter size)** | **Binomial part (breeding proba)** | **AIC** | **loglik** | **npar** | **deltaAIC** |
| --- | --- | --- | --- | --- | --- | --- |
| 1 | PCA2:BL | PCA2:BC + PCA2:Pprod2week + PCA2:Temp + PCA2:Ab + PCA1:BC + PCA1:Ab + PCA1:PCA2 | 4408.33866788858 | -2183.16933394429 | 21 | 0 |
| 2 | 1 | PCA2:BC + PCA2:Pprod2week + PCA2:Temp + PCA2:Ab + PCA1:BC + PCA1:Ab + PCA1:PCA2 | 4408.59869119334 | -2184.29934559667 | 20 | 0.26002330475967 |
| 3 | PCA1:PCA2 | PCA2:BC + PCA2:Pprod2week + PCA2:Temp + PCA2:Ab + PCA1:BC + PCA1:Ab + PCA1:PCA2 | 4409.4074805393 | -2182.70374026965 | 22 | 1.06881265072025 |
| 4 | PCA2:BL | PCA2:BC + PCA2:Pprod2week + PCA2:Temp + PCA2:Ab + PCA1:BC + PCA1:Temp + PCA1:Ab + PCA1:PCA2 | 4409.49065039301 | -2182.7453251965 | 22 | 1.15198250442972 |
| 5 | PCA2:Pprod2week | PCA2:BC + PCA2:Pprod2week + PCA2:Temp + PCA2:Ab + PCA1:BC + PCA1:Ab + PCA1:PCA2 | 4409.5141221436 | -2183.7570610718 | 21 | 1.17545425501976 |
| 6 | PCA2:BL + PCA2:Past Ab | PCA2:BC + PCA2:Pprod2week + PCA2:Temp + PCA2:Ab + PCA1:BC + PCA1:Ab + PCA1:PCA2 | 4409.58575295201 | -2182.79287647601 | 22 | 1.24708506342995 |
| 7 | PCA2:BL + PCA1:PCA2 | PCA2:BC + PCA2:Pprod2week + PCA2:Temp + PCA2:Ab + PCA1:BC + PCA1:Ab + PCA1:PCA2 | 4409.71653763681 | -2181.85826881841 | 23 | 1.3778697482303 |
| 8 | PCA2:BL + PCA2:Pprod2week | PCA2:BC + PCA2:Pprod2week + PCA2:Temp + PCA2:Ab + PCA1:BC + PCA1:Ab + PCA1:PCA2 | 4409.71998655794 | -2182.85999327897 | 22 | 1.38131866935964 |
| 9 | 1 | PCA2:BC + PCA2:Pprod2week + PCA2:Temp + PCA2:Ab + PCA1:BC + PCA1:Temp + PCA1:Ab + PCA1:PCA2 | 4409.75067369776 | -2183.87533684888 | 21 | 1.4120058091803 |
| 10 | PCA2:BL | PCA2:BC + PCA2:Pprod2week + PCA2:Temp + PCA2:Ab + PCA1:BC + PCA1:Pprod2week + PCA1:Ab + PCA1:PCA2 | 4409.78042251801 | -2182.89021125901 | 22 | 1.44175462942985 |
| 11 | PCA2:Pprod2week + PCA1:PCA2 | PCA2:BC + PCA2:Pprod2week + PCA2:Temp + PCA2:Ab + PCA1:BC + PCA1:Ab + PCA1:PCA2 | 4409.7968147359 | -2181.89840736795 | 23 | 1.45814684731977 |
| 12 | 1 | PCA2:BC + PCA2:Pprod2week + PCA2:Temp + PCA2:Ab + PCA1:BC + PCA1:Pprod2week + PCA1:Ab + PCA1:PCA2 | 4410.04044582277 | -2184.02022291138 | 21 | 1.70177793419043 |
| 13 | PCA2:BL | PCA2:BC + PCA2:Pprod2week + PCA2:Temp + PCA2:Ab + PCA1:BC + PCA1:Ab + PCA1:PCA2 | 4410.13718600405 | -2183.06859300202 | 22 | 1.79851811546996 |
| 14 | PCA2:BL | PCA2:BC + PCA2:Pprod2week + PCA2:Temp + PCA2:Ab + PCA1:BL + PCA1:BC + PCA1:Ab + PCA1:PCA2 | 4410.28914652284 | -2183.14457326142 | 22 | 1.95047863426043 |
| 15 | PCA2:Past Ab | PCA2:BC + PCA2:Pprod2week + PCA2:Temp + PCA2:Ab + PCA1:BC + PCA1:Ab + PCA1:PCA2 | 4410.29925518101 | -2184.1496275905 | 21 | 1.96058729243032 |
| 16 | PCA2:BL | PCA2:BL + PCA2:BC + PCA2:Pprod2week + PCA2:Temp + PCA2:Ab + PCA1:BC + PCA1:Ab + PCA1:PCA2 | 4410.33866077291 | -2183.16933038645 | 22 | 1.99999288432991 |
| 17 | 1 | PCA2:BC + PCA2:Pprod2week + PCA2:Temp + PCA2:Ab + PCA1:BC + PCA1:Ab + PCA1:PCA2 | 4410.43452113793 | -2184.21726056897 | 21 | 2.0958532493496 |
| 18 | 1 | PCA2:BC + PCA2:Pprod2week + PCA2:Temp + PCA2:Ab + PCA1:BL + PCA1:BC + PCA1:Ab + PCA1:PCA2 | 4410.5491698276 | -2184.2745849138 | 21 | 2.21050193902011 |
| 19 | PCA1:PCA2 | PCA2:BC + PCA2:Pprod2week + PCA2:Temp + PCA2:Ab + PCA1:BC + PCA1:Temp + PCA1:Ab + PCA1:PCA2 | 4410.55946304373 | -2182.27973152186 | 23 | 2.22079515514997 |
| 20 | 1 | PCA2:BL + PCA2:BC + PCA2:Pprod2week + PCA2:Temp + PCA2:Ab + PCA1:BC + PCA1:Ab + PCA1:PCA2 | 4410.59868407767 | -2184.29934203883 | 21 | 2.26001618909049 |
| 21 | PCA2:BL + PCA2:Pprod2week + PCA1:PCA2 | PCA2:BC + PCA2:Pprod2week + PCA2:Temp + PCA2:Ab + PCA1:BC + PCA1:Ab + PCA1:PCA2 | 4410.64993492223 | -2181.32496746112 | 24 | 2.31126703365044 |
| 22 | PCA2:BL | PCA2:BC + PCA2:Pprod2week + PCA2:Temp + PCA2:Ab + PCA1:BC + PCA1:Pprod2week + PCA1:Temp + PCA1:Ab + PCA1:Season_co | 4410.65525671441 | -2182.3276283572 | 23 | 2.31658882582997 |
| 23 | PCA2:Pprod2week | PCA2:BC + PCA2:Pprod2week + PCA2:Temp + PCA2:Ab + PCA1:BC + PCA1:Temp + PCA1:Ab + PCA1:PCA2 | 4410.66610464803 | -2183.33305232402 | 22 | 2.32743675945039 |
| 24 | PCA2:BL + PCA2:Past Ab | PCA2:BC + PCA2:Pprod2week + PCA2:Temp + PCA2:Ab + PCA1:BC + PCA1:Temp + PCA1:Ab + PCA1:PCA2 | 4410.73773545644 | -2182.36886772822 | 23 | 2.39906756785967 |
| 25 | PCA2:BL | PCA2:BC + PCA2:Pprod2week + PCA2:Temp + PCA2:Ab + PCA1:BC + PCA1:Ab | 4410.74644069806 | -2185.37322034903 | 20 | 2.40777280947987 |
